# Change in brain activity through virtual reality-based brain-machine communication in a chronic tetraplegic subject with muscular dystrophy

**DOI:** 10.1186/1471-2202-11-117

**Published:** 2010-09-16

**Authors:** Yasunari Hashimoto, Junichi Ushiba, Akio Kimura, Meigen Liu, Yutaka Tomita

**Affiliations:** 1 School of Fundamental Science and Technology, Graduate School of Science and Technology, Keio University, Kanagawa, Japan; 2 Department of Biosciences and Informatics, Faculty of Science and Technology, Keio University, Kanagawa, Japan; 3 Keio University Tsukigase Rehabilitation Center, Shizuoka, Japan; 4 Department of Rehabilitation Medicine, Keio University School of Medicine, Tokyo, Japan

## Abstract

**Background:**

For severely paralyzed people, a brain-computer interface (BCI) provides a way of re-establishing communication. Although subjects with muscular dystrophy (MD) appear to be potential BCI users, the actual long-term effects of BCI use on brain activities in MD subjects have yet to be clarified. To investigate these effects, we followed BCI use by a chronic tetraplegic subject with MD over 5 months. The topographic changes in an electroencephalogram (EEG) after long-term use of the virtual reality (VR)-based BCI were also assessed. Our originally developed BCI system was used to classify an EEG recorded over the sensorimotor cortex in real time and estimate the user's motor intention (MI) in 3 different limb movements: feet, left hand, and right hand. An avatar in the internet-based VR was controlled in accordance with the results of the EEG classification by the BCI. The subject was trained to control his avatar via the BCI by strolling in the VR for 1 hour a day and then continued the same training twice a month at his home.

**Results:**

After the training, the error rate of the EEG classification decreased from 40% to 28%. The subject successfully walked around in the VR using only his MI and chatted with other users through a voice-chat function embedded in the internet-based VR. With this improvement in BCI control, event-related desynchronization (ERD) following MI was significantly enhanced (*p *< 0.01) for feet MI (from -29% to -55%), left-hand MI (from -23% to -42%), and right-hand MI (from -22% to -51%).

**Conclusions:**

These results show that our subject with severe MD was able to learn to control his EEG signal and communicate with other users through use of VR navigation and suggest that an internet-based VR has the potential to provide paralyzed people with the opportunity for easy communication.

## Background

For severely paralyzed people or subjects in a "locked-in" state, direct brain-machine interaction provides a way of re-establishing communication. Electroencephalogram (EEG)-based brain-computer interfaces (BCIs) recognize intention-induced changes in ongoing EEG signals and translate different mental states into the appropriate commands for operating consumer electronics or spelling devices. To provide a natural interaction between humans and machines, BCIs must constantly classify ongoing electric cortical activity and allow users real-time control of external devices. Users should know exactly how the ongoing electric cortical activity is changing, and hence, considerable feedback must be provided.

Virtual reality (VR) has been used as information-rich feedback for BCIs [1-8]. The precise three-dimensional graphics in VR are believed to help users feel a sense of reality during BCI control. We developed a BCI that classifies 3 different types of motor intentions (MIs) on the basis of the self-paced operation of an avatar (the user's graphical self-representation). The experiment was done not only to check the effectiveness of long-term use of BCI with VR feedback but also to deliver e-communication opportunities to paralyzed persons through the internet. For simplicity, only 3 bipolar EEG channels were used for the classification. Using our portable VR-based BCI system, a tetraplegic subject with muscular dystrophy (MD) successfully communicated with others over a 5-month period. We report this single case to demonstrate our experience with the feasibility of internet-based VR-BCI as a new communication tool. We also describe plastic changes in EEG topography by long-term BCI training in the MD subject.

## Results

### Classification and error rate over time

With the increase in training days, the subject decreased the error rate of the classification and finally succeeded in controlling his avatar and communicating with other users who were logged in to Second Life^® ^from elsewhere (the QuickTime demonstration film is available at http://www.bme.bio.keio.ac.jp/01news/). Movie files are also available. Additional files 1 and 2 show the demonstration in the initial and final conditions of avatar controlling, respectively.

**Figure 1:**
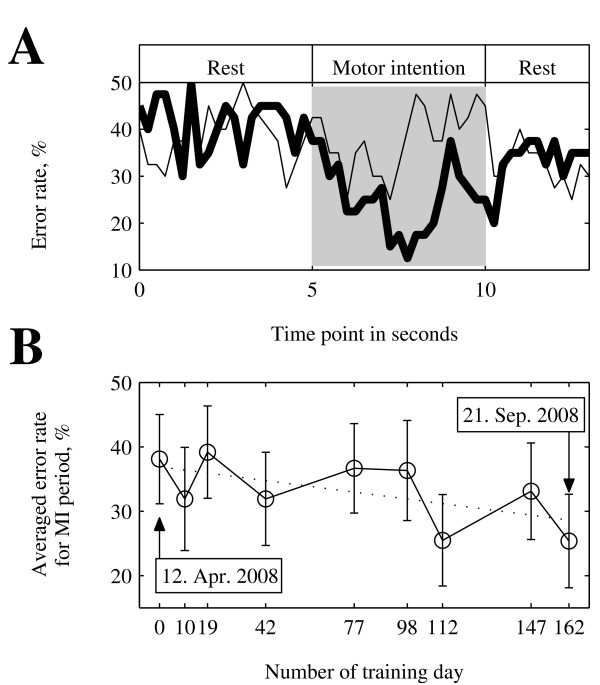
**Changes in the error rate for classification between left- and right-hand motor intentions (MIs)**. (A) The error rate over time during cue-paced sessions on the initial (thin line) and final (thick line) training days. The gray shaded area indicates the duration of the left- or right-hand MI. (B) The change in average error rate during the MI period (gray shaded area in panel A) for a total of 9 training days.

The error rate in the classification of left- and right-hand MI decreased after the avatar-control training (Fig. 1). On the final training day, about 5 months after admission, the error rate showed a larger decrease during the MI period after the cue than on the initial day (Fig. 1A). Fig. 1B shows the average error rate during MI for all 9 days of the training. The average error rate reduced from about 38% to 25%.

Because of the subject's health condition, we could not conduct all experiments at a constant interval of 2 weeks. We started the training on 12 Apr 2008 and ended it on 21 Sep 2008 (162 days; approximately 5 months). The other 7 experimental dates are indicated on the x-axis scale in Fig. 1.

**Figure 2:**
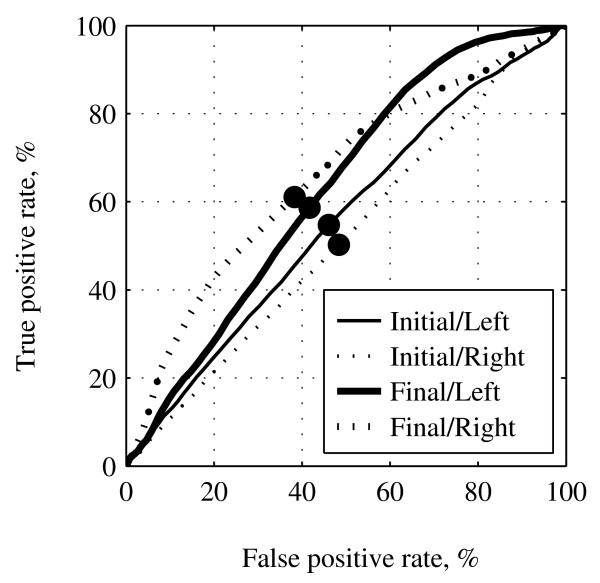
**Receiver operating characteristic (ROC) curve for left- and right-hand MI detection in the initial and final training sessions**. The thin curves are the data in the initial training sessions and the thick curves are the data in the final training sessions for the left- (solid line) and right-hand MI (dotted line). The circled points on the curves mark the point of equal balance of sensitivity and selectivity.

**Figure 3:**
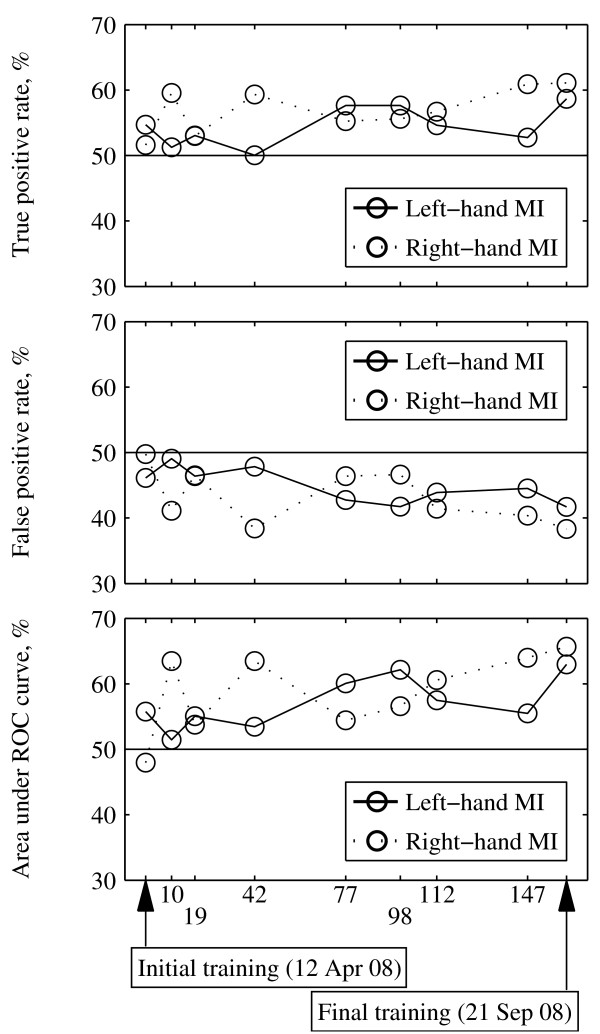
**Change in true positive rate (TPR), false positive rate (FPR), and area under the ROC curve (AUC) during the avatar-control training period**. The lines indicate the change of TPR (A), FPR (B), and AUC (C) for a total of 9 training days. The TPR and FPR values corresponding to the best threshold for the solid ROC curves shown in Fig. 2 was extracted. The solid lines represent the left-hand IM and the dotted lines represent the right-hand MI.

Along with decreasing the classification errors, the subject also improved his true positive rate (TPR) and false positive rate (FPR) through the training. Fig. 2 shows the receiver operating characteristic (ROC) curves for left- and right-hand MI detection in the initial and final training sessions and Fig. 3 indicates changes in TPR, FPR, and area under the ROC curve (AUC) during the BCI training period. These 3 parameters tended to increase.

### Multichannel EEG recording

**Figure 4:**
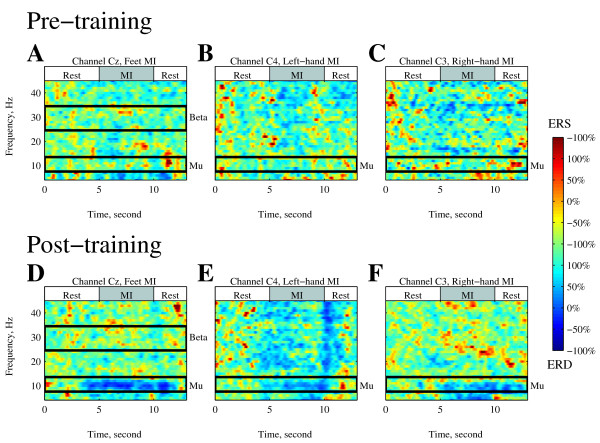
**ERD and ERS following the 3 MIs computed from Channels Cz, C4, and C3**. The 3 columns indicate the 3 kinds of MI (feet MI: A and D; left-hand MI: B and E; right-hand MI: C and F). (A-F) Time-frequency maps in 4-45 Hz. Panels A-C represent the pre-training day and D-F represent the post-training day.

**Figure 5:**
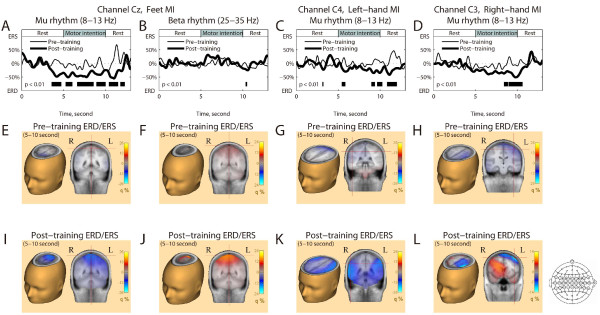
**Topographies following the 3 MIs computed from 27-channel EEG data**. (A-D) ERD/ERS for time points in the 8-13 Hz bands for all MIs and in the 25-35 Hz bands for feet MI. The thin curves are the data on the pre-training day and the thick curves are the data on the post-training day. The horizontal gray bar on the time axis indicates the duration of the MI and rest tasks. The filled bars at the bottom of the panel indicate significant differences of ERD/ERS between the 2 days (Wilcoxon signed-rank test, *p *< 0.01). (E-L) Topographical ERD/ERS maps on the pre- and post-training days. The magnitude of ERD/ERS was extracted from the 8-13 Hz and 25-35 Hz bands during 5.0-10.0 s. The set of electrodes used for these maps is shown to the right of panel L.

Figs 4 and 5 represent the event-related desynchronization (ERD)/event-related synchronization (ERS) during the 27-channel EEG recording. Fig. 4A-4F shows the time-frequency maps of the ERD/ERS on both the pre- and post-training days. The mu ERD (8-13 Hz) values following the feet, left-hand, and right-hand MIs were all enhanced by the training (Fig. 5A, C, and 5D). The most obvious change occurred at Cz for the feet MI (approximately -29% to -55%; Fig. 5A). The peak of ERD at C4 for the left-hand MI (from -23% to -42%; Fig. 5C) and at C3 for the right-hand MI (from -22% to -51%; Fig.5D) also increased. These ERD changes showed a significant difference (*p *< 0.01) between the pre- and post-training during the MI period (5.0-10.0 s). Whereas the subject controlled the avatar using the BP of the beta frequency band (25-35 Hz) at Cz, the enhancement of the beta ERS showed no significant difference (from 21% to 31%; Fig. 5B) during the MI period.

EEG topographies on the pre- (Figs. 5E to 5H) and post-training days (Figs. 5I to 5L) show the changes in ERD/ERS somatotopic organization. In the post-training days, mu ERD and beta ERS following the feet MI was observed at the vertex and the ERD values following the left- and right-hand MI were at the right and left hemispheres, respectively. Incidentally, in the left-hand MI, bilateral mu ERD was observed.

In addition, the changes in the TPR and FPR for all the three MIs (feet, left hand, and right hand) through the pre- and post-training sessions are shown in Table 1.

**Table 1:** Summary of true positive rate (TPR), false positive rate (FPR), and area under the ROC curve (AUC) on the pre- and post-training days.

		TPR(%)	FPR(%)	AUC(%)
Feet MI	Pre-training	55	47	55
	Post-training	56	41	59

Left hand training	Pre-training	54	46	56
	Post-training	61	49	59

Right hand training	Pre-training	53	49	53
	Post-training	50	45	54

## Discussion

In this study, we developed an MI-based asynchronous BCI system that can help users navigate an avatar in an internet-based VR. This study was the first to show the feasibility of internet-based virtual reality (Second Life^®^) combined with BCI for realization of social communication.

Currently, the BCI with the highest degree of freedom in BCIs for VR navigation is published by Scherer et al. (2008), a system that classifies EEG signals and translates the classification results into 4 commands: forward movement, left rotation, right rotation, and stop [1], similar to our BCI. Considering the degree of freedom, other VR navigation systems with BCI has fewer commands than the BCI by Scherer et al. In 2006, Pfurtscheller et al. published a BCI with 3 commands: forward movement, backward movement, and stop [2], and most of the other relevant studies showed BCIs that control 2 commands [3-5].

To move about freely in VR, 4 commands comprise the minimum degree of freedom. Implementation of this system achieved the result of physically challenged movement in the VR at free will. In addition, our system's use of Second Life^®^, the VR that the voice chat function through the Internet is embedded in, helps the physically challenged communicate with others. The subject in our study successfully walked and chatted with other VR users while using the BCI at home. Networked VR on the Internet would create synergy in terms of assistive technology for the physically challenged.

A physically challenged individual with MD who was trained to use this system changed his EEG pattern through long-term BCI use. It is notable that the subject, who had not moved his feet in approximately 30 years, still had the ability to change the EEG patterns to follow the feet MI in the feet representation area. Moreover, his hands are also severely impaired, but we found that his hand MIs evolved EEG changes. Since MD causes progressive changes in neuromuscular properties over the course of months or years, it was unclear whether long-term BCI use actually changed EEG patterns and classification accuracy in the MD subject.

Our novel finding in this study is the plastic changes of EEG activity by long-term BCI training in the MD subject. Although the Graz BCI group has also published a long-term BCI training study in a patient [9], that particular patient had injured his spinal cord. Spinal cord injuries (SCI) cause myelopathy or damage to nerve roots or myelinated fiber tracts that carry signals to and from the brain and are categorized as traumatic injuries. Since the site responsible for functional loss is at the spinal cord, the brain itself is intact. Indeed, another study indicated that motor skills were considerably intact in SCI patients [10]. Therefore, patients with SCI are very likely capable of using BCI. On the other hand, MD shows progressive skeletal muscle weakness, and the neural strategy of motor control might change over the course of several months or years. Thus, it is more unclear in MD than in SCI whether long-term BCI training positively affects EEG change for BCI control. The present study is the first to report half-year BCI use by a patient with MD and evaluate changes in EEG and BCI accuracy. While ERD/ERS caused by MI became more prominent over the study duration of a few months, the neuromuscular properties of patients with MD change over the course of months or years. From such observed post-training ERD changes in each representation area, we speculate that sensorimotor cortex plasticity was one of the factors causing the BCI accuracy improvement.

## Conclusions

We reported the use that 1 severely paralyzed individual made of our original BCI system. We observed changes in ERD and ERS patterns and increases in BCI performance over long-term use of this system. Our results suggest that it is possible to develop VR systems that allow severely paralyzed patients to communicate with others in a virtual world in the same way as a healthy person.

## Methods

### Subject

The tetraplegic subject of this study was a 41-year-old man who has suffered from severe MD for more than 30 years. His active ranges of motion in the shoulders, elbows, wrists, and fingers are 0 degrees, except for 30 degrees in forearm supination and 25 degrees in thumb carpometacarpal abduction. He can barely bend his fingers so he cannot use an ordinary mouse or keyboard. He is dependent on trunk support to maintain a sitting position but has no difficulty speaking.

**Figure 6:**
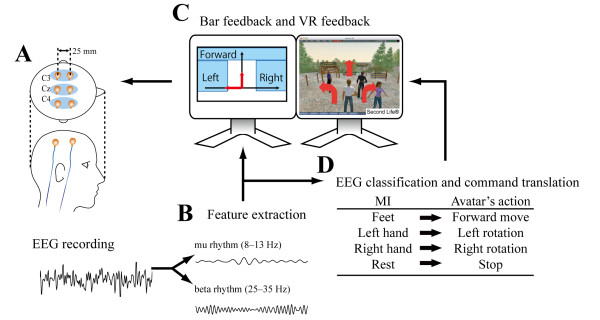
**Schematic diagram of the virtual reality (VR)-based brain-computer interface for avatar control**. (A) Three pairs of bipolar EEG recordings. The electrode placements were at Cz, C4, C3, and 25 mm anterior of these positions. (B) Feature extraction. Band power (BP) in the subject-specific frequency bands (mu rhythm: 8-13 Hz; beta rhythm: 25-35 Hz) was extracted from the ongoing EEG signals. (C) Visual feedback. The BP feature of the beta rhythm (25-35 Hz) at Cz and the output of the LDA classifier for hand MI classification are displayed in the left-hand screen as vertical and horizontal feedback bars, respectively. The internet-based VR application is displayed in the right-hand screen. (D) Command translation. The user's motor intention was translated into the avatar's action.

Informed consent for this study was given by the subject before the experiments. The experimental protocol was approved by the local ethics committee of Keio University. The subject participated in the experiment every 2 weeks over a 5-month period. We conducted a total of 9 days of avatar-control training using our VR-based BCI (Fig. 6). In addition, to evaluate the cortical activity changes caused by the training, we conducted multichannel monopolar EEG recordings on both pre- and post-training days. Every experiment was finished within 2 hours to avoid tiring the subject.

### EEG recording

EEG signals were recorded with 3 bipolar surface electrodes named channel Cz, channel C4, and channel C3 (anodes: Cz, C4, and C3 of the international 10/20 electrode system; cathodes: 25 mm anterior to respective anodes; see also Fig. 6A), band-pass filtered in the frequency range from 5 to 100 Hz and digitized at 256 Hz by a biosignal acquisition system (g.USBamp, Guger Technologies, Graz, Austria). The ground electrode was positioned on the subject's forehead.

### Feature extraction

EEG contains different specific frequency bands (e.g., standard mu [8-13 Hz] or beta [18-40 Hz] bands) that are particularly important for classifying different brain states and especially for discriminating what a subject is imagining. To extract this information, we computed the spectral power in the reactive frequency bands ("band power" [BP] [11]) as the subject's EEG feature. The procedure for calculating BP is described below.

Channels Cz, C4, and C3 were analyzed by calculating time-frequency maps of EEG data recorded on a pre-training day (the recording details are described later). Based on these maps, the most reactive frequency bands (8-13 Hz for the mu rhythm 25-35 Hz for the beta rhythm) were manually selected by visual inspection and used to set up the 2 classifiers. These procedures were also described in another study [12]. These reactive bands were used both for online processing of the BCI and for offline analysis of subject-specific mu and beta rhythms.

The BP of each mu and beta rhythm was estimated from ongoing EEG by digital band-pass filtering with a Butterworth filter of order 4, squaring, and averaging the samples over 1 s. The processing was done sample by sample. Afterward, the logarithm was computed from these processed signals (Fig. 6B). Other studies of healthy subjects reported that these rhythms somatotopically change in amplitude by motor imagery of the feet or hands [13,14]. We expected that similar EEG patterns would be observed during MI in the tetraplegic subject. According to somatotopy, the feet MI was detected using the BP in the beta frequency band of the foot representation area (channel Cz) and the hand MI was detected using the BP of the mu and beta rhythms in the hand representation area (channels C4 and C3).

### Classification and error rate over time

We designed the BCI to detect the feet MI with a single threshold detector because the beta rhythm at Cz produces increases that can be detected by a single detector [15]. On the other hand, the left- and right-hand MI were detected by 2 threshold detectors and Fisher's linear discriminant analysis (LDA) [16] because in certain cases the mu and beta rhythms at C4 and C3 produce only small asymmetric changes by unilateral hand MIs.

A cue-paced session for the initial setting of the LDA classifier was performed without feedback prior to the avatar-control training. The protocol was proposed by Guger et al. [17]. The session consisted of 40 trials. Each trial started with presentation of a fixation cross at the centre of the computer screen for 4 s to allow the subject to pay attention prior to the guidance of the motor tasks. Visual guidance of a left- or right-hand MI task was then displayed for 1 s and the subject was asked to imagine the directed movement for 5 s. Finally, an inter-trial interval with a blank screen followed for 3 s before the next trial. A left- or right-hand MI task was randomly requested with 20 trials for each MI.

The BP of each mu and beta rhythm in channels C3 and C4 was calculated during this time and an LDA was performed for these 4 features at every 250 ms (4 Hz) within the trial interval (0-13 s) rather than sample by sample (256 Hz) to enable rapid analysis at these time points [5,18,19]. For every time point, a feature vector containing 4 elements (mu BP at channels C3 and C4 and beta BP at channels C3 and C4) was generated. Hence, we had 40 vectors (since one session had 40 trials) at each time point with an annotation of either left-hand or right-hand intention. At each time point, the 40 feature vectors were used to set the LDA parameters and to calculate the error rate of the classification by 10-fold cross-validation as follows. Forty vectors for a certain time point were mixed randomly and divided into 10 small packets, each with 4 vectors. Then, 9 packets were used as training data to set the LDA parameters and the remaining packet was classified by the LDA. This operation was done 10 times with different pairs of packets, and we averaged the 10 error rates and 10 sets of LDA parameters. This calculation was performed at every time point so the classification error rate over time was obtained. The LDA parameter at the time point with the minimum error rate was used for online classification of the EEG in the avatar-control training.

Importance in self-paced (at free will) BCI operation is not only the rate of errors during the MI periods (true positive rate: TPR) but also the rate of errors during the rest periods (false positive rate: FPR). We calculated the TPR and FTR with the same method used in another study [20] for feet, left-hand, and right-hand MI detection by sample-by-sample analysis and briefly explain the method below.

In this analysis, we divided the output signals of classifiers into MI periods (events) and rest periods (non-events). The two axes of the ROC curves were TPR and FPR. The former is a measure of sensitivity while the latter is a measure of electivity. These quantities are captured by the following:

$$\begin{array}{l} R_{TP}=N_{TP}/(N_{TP}+N_{FN})\\ R_{FP}=N_{FP}/(N_{TN}+N_{FP}) \end{array}$$

where *R*_*TP *_*and R*_*FP *_are TPR and FPR, respectively. *N*_*TP*_, *N*_*FP*_, *N*_*TN*_, and *N*_*FP *_are the number of true positives, false negatives, true negatives, and false positives, respectively. Note that all of these values are counted in the samples. The area under the ROC curve (AUC) varies between 0 and 1 and gives a measure of the reparability of 2 classes with an area of 1 for complete separation.

From each ROC curve, a threshold corresponding to the point of the ROC curve closest to the line *R*_*TP *_= 1- *R_FP _*is selected as an indication of equal balance between TPR and FPR.

The cue-paced feet MI task was not conducted in the avatar-control training period (details are described later) since only a simple threshold detected the feet MI and we did not calibrate the detector with an EEG signal from the cue-paced session. Therefore, the changes in TPR and FPR during the training period were analyzed for only left-hand and right-hand MIs. Analysis of all 3 types of MI (feet, left hand, and right hand) was performed of the pre- and post-training sessions.

### Avatar-control training in VR

After setting the LDA classifier, we performed the avatar-control training. Both the beta BP at channel Cz and the output of the LDA classifier for hand MI classification were converted to feedback bars in real time (via asynchronous BCI [21]; see also Fig. 3C). When the feedback bars exceeded the thresholds that were manually set at the beginning of the training, a keyboard command signal corresponding to the MI type was sent to the computer running the VR system. Feet, left-hand, and right-hand MIs were translated into 3 distinct actions for the avatar: going forward, left rotation, and right rotation, respectively (Fig. 3D). No command was sent when the feedback bar was below the threshold. The 2 classifiers for feet and hand MIs ran in parallel, e.g., to make it possible for the avatar to move forward and turn at same time. The display of the bar length and the avatar's action was updated at a rate of 16 times per second. We used Second Life^® ^(Linden Lab, San Francisco, CA, USA) as the Internet-based VR in our BCI. Second Life^® ^is an accessible three-dimensional VR in which users can interact via their avatars and talk to each other using the incorporated typing or voice-chat system.

The subject was trained to control his avatar in Second Life^® ^by entering virtual buildings or walking with other avatars using our BCI for about 1 hour on each training day. To check the improvement of classification accuracy, we conducted the cue-paced session again after the training.

### Multichannel EEG recording

We conducted multichannel EEG recordings on both pre- and post-training days to evaluate the topographic changes caused by the avatar-control training. The EEG was referenced to the right earlobe and recorded from 27 Ag/AgCl scalp electrodes placed close to the sensorimotor area (FT7, FC5, FC3, FC1, FCz, FC2, FC4, FC6, FT8, T7, C5, C3, C1, Cz, C2, C4, C6, T8, TP7, CP5, CP3, CP1, CPz, CP2, CP4, CP6, and TP8 of the international 10/20 electrode system). The monopolar EEG was amplified and filtered with 5-100 Hz band-pass filters. The apparatus, filter setting, and sampling rate were the same as those used for the avatar-control training.

Cue-paced sessions for the multichannel recording were performed. The requested MIs were the feet, left-hand, and right-hand in a random order. The recording was divided into 5 sessions consisting of 30 trials each, which led to 50 repetitions for each MI task. There were sufficient breaks between the sessions to prevent fatigue. The timing of the visual guidance and the length of each trial were the same as in the cue-paced session for the initial LDA setting.

### Analysis of multichannel EEG

To quantify the impact of the training on the BP, we computed time-frequency maps using the data on pre- and post-training days as follows. First, the monopolar EEG signals were converted to a reference-free form by a Laplacian algorithm [22] taking the difference between the potentials at an electrode of interest and the mean of its four nearest-neighbor electrodes (e.g., for electrode Cz, these were FCz [anterior], C1 [left], C2 [right], and CPz [posterior]). This spatial filter was used to retain a high signal-to-noise ratio [23]. Second, the power of the filtered signals at Cz, C4, and C3 were computed with a fast Fourier transform by following the segmentation of the data stream in approximately 94% overlapped segments of 1-s duration, resulting in a 1 Hz frequency resolution, and averaged separately for the 3 conditions (feet, left-hand, and right-hand MI). Third, the BP of every 1 Hz in 4-45 Hz was averaged for a rest period (0.0-4.0 s of the trials) and assigned to 0% according to the following formula: ERD/ERS = [(power during rest) - (power during experimental condition)]/(power during rest). These decreases (negative values) and increases (positive values) are the so-called ERD and ERS [24]. The values of the ERD/ERS calculated by the above formula were then separately averaged for each mu and beta band. Afterward, the Wilcoxon signed-rank test was applied for a comparison of the pre- and post-training days. The calculation procedure described above is the power method of Kalcher and Pfurtscheller [25].

Topographical ERD/ERS maps for the subject-specific frequency bands were shown via projection onto a standard brain template using the Brain Electrical Source Analysis Software Version 5.1.8 (BESA, MEGIS Software GmbH, Gräfelfing, Germany). To show the distribution of ERD/ERS over the whole head, EEG signals at all 27 channels were used.

## Authors' contributions

YH made substantial contributions to the experimental conception and design, data acquisition, and data analysis and interpretation; JU was involved in drafting and revising the manuscript; and AK, ML, and YT gave final approval of the revised manuscript for publication. All authors read and approved the final manuscript.

## Supplementary Material

Additional file 1 **Demonstration movie recorded during the initial period of the avatar-control training**. In spite of the subject's intention to enter a training course (green path) in the VR (virtual reality), he proceeded in another unintended direction. It can be seen that he wanders in the area with no success of going back to his desired direction. Click here for file

Additional file 2 **Demonstration movie recorded during the final period of the avatar-control training**. The window consists of two sub-windows on the right-hand and the image of computer monitor on the left-hand. The lower right sub-window shows the subject trying to control the avatar in the monitor. The upper right sub-window depicts the volunteer students participated in this demonstration. They logged in the VR from their university located 15 km away from the subject's home. The subject first tries to reach one of the volunteers' avatars. Although he approaches another avatar accidentally at the beginning, he managed to correct his way and successfully reached the avatar that he intended at first. Other volunteers' avatars consequently came towards the subject's avatar and celebrated the subject's success by cheering, dancing and chapping. Click here for file
